# Altered Functional Connectivity in White and Gray Matter in Patients With Multiple Sclerosis

**DOI:** 10.3389/fnhum.2020.563048

**Published:** 2020-12-02

**Authors:** Jing Huang, Muwei Li, Qiongge Li, Zhipeng Yang, Bowen Xin, Zhigang Qi, Zheng Liu, Huiqing Dong, Kuncheng Li, Zhaohua Ding, Jie Lu

**Affiliations:** ^1^Xuanwu Hospital, Capital Medical University, Beijing, China; ^2^Institute of Imaging Science, Vanderbilt University, Nashville, TN, United States; ^3^Department of Computer Science, Chengdu University of Information Technology, Chengdu, China; ^4^School of Computer Science, Faculty of Engineering, The University of Sydney, Sydney, NSW, Australia

**Keywords:** white matter, functional connectivity, resting-state, MRI, multiple sclerosis

## Abstract

**Background**: Functional magnetic resonance imaging (fMRI) has been widely used to assess neural activity changes in gray matter (GM) in patients with multiple sclerosis (MS); however, brain function alterations in white matter (WM) relatively remain under-explored.

**Purpose**: This work aims to identify the functional connectivity in both the WM and the GM of patients with MS using fMRI and the correlations between these functional changes and cumulative disability as well as the lesion ratio.

**Materials and Methods**: For this retrospective study, 37 patients with clinically definite MS and 43 age-matched healthy controls were included between 2010 and 2014. Resting-state fMRI was performed. The WFU Pick and JHU Eve atlases were used to define 82 GM and 48 WM regions in common spaces, respectively. The time courses of blood oxygen level-dependent (BOLD) signals were averaged over each GM or WM region. The averaged time courses for each pair of GM and WM regions were correlated. All 82 × 48 correlations for each subject formed a functional correlation matrix.

**Results**: Compared with the healthy controls, the MS patients had a decreased temporal correlation between the WM and the GM regions. Five WM bundles and four GM regions had significantly decreased mean correlation coefficients (CCs). More specifically, the WM functional alterations were negatively correlated with the lesion volume in the bilateral fornix, and the mean GM-averaged CCs of the WM bundles were inversely correlated with the lesion ratio (*r* = −0.36, *P* = 0.012). No significant correlation was found between WM functional alterations and the paced auditory serial addition test score, Expanded Disease Severity Scale score, and Multiple Sclerosis Severity Score (MSSS) in MS.

**Conclusions**: These findings highlight current gaps in our knowledge of the WM functional alterations in patients with MS and may link WM function with pathological mechanisms.

## Introduction

Multiple sclerosis (MS) is a demyelinating disease characterized by focal and diffused damage over space and time (Reich et al., 2018). In the past decade, functional magnetic resonance imaging (fMRI) has been widely used in patients with MS to detect the neural connectivity of the cortex in both task-based and resting states (Rocca et al., 2005; Roosendaal et al., 2010). In recent years, graph theory has been adopted as a novel approach, enabling researchers to model brain networks, identify functional connectivity patterns under various conditions, and evaluate the topological characteristics of brain networks (Azarmi et al., 2019). Miri et al. (2019) elaborated on the evaluation of the graph structure in patients with MS, which is helpful for distinguishing the disrupted functional topology in patients with early-stage MS presenting with cognitive impairment. fMRI is a potentially powerful tool to understand neural plasticity and adaptive reorganization. However, studies considering only gray matter (GM) function have limitations because MS affects both white matter (WM) and GM tissue compartments (Wattjes et al., 2015). If we study the functions of both WM and GM, we will obtain a better understanding of the physiological mechanism of MS. Therefore, given that the changes in WM function in MS are completely unknown, this topic needs to be explored.

A primary reason for the lack of fMRI studies of WM is that the vascular density in WM is much lower than that in GM, and thus the WM-based blood oxygen level-dependent (BOLD) signals are weaker, leading to a lack of understanding of WM BOLD signals by nearly the entire research community (Gawryluk et al., 2014). It has recently been observed that BOLD signals exhibit similar temporal and spectral profiles in both WM and GM, suggesting that WM BOLD signals may also encode neural activities that are detectable with appropriate methods (Marussich et al., 2017; Peer et al., 2017; Huang et al., 2018). In particular, by parcellating brain WM and GM into sets of segregated regions and identifying pairwise temporal correlations between the WM and GM regions, Ding et al. (2018) observed that WM BOLD signals were implicated in neural activities.

Due to the lack of postsynaptic potentials, which are considered the main source of BOLD signals, the reliability of WM BOLD signals in resting-state fMRI remains controversial. Additionally, the biophysical origins of BOLD signals in WM remain unclear. Nevertheless, Wu et al. (2019) used a non-human primate model to study the resting-state connectivity patterns between cortical volume and specific WM bundles. The resting-state BOLD signal correlations between the WM bundles and the GM regions they connected with were found to be directly related to the anatomical structure and density of the WM fibers. A recent study (Li et al., 2019) has also successfully measured the hemodynamic response function in specific WM tracts using MRI. These developments in fMRI studies of WM potentially provide a new avenue for brain research, such that novel insights into structure–function relations may be derived. By taking full advantage of the technical concepts proposed by Ding et al. (2018), to the best of our knowledge, the present study is the first to explore WM functional alterations and associated GM functional changes in patients with MS. Specifically, we examined the regional distribution and extent of WM functional impairment and investigated how the WM functional alterations were related to clinical performance and the WM lesion load. We hypothesized that: (i) functional alterations in WM can be detected in patients with MS; (ii) WM functional abnormalities are associated with clinical characteristics; and (iii) we preliminarily investigated the association between WM functional changes and the quantitative WM lesion load.

## Materials and Methods

### Ethical Approval and Patient Consent

This retrospective study was approved by the institutional review board of Xuanwu Hospital, Capital Medical University, and written informed consent was obtained from all participants.

### Participants

Thirty-seven patients with relapsing–remitting MS (26 females and 11 males) were recruited between 2010 and 2014. The eligibility criteria were patients with a relapsing–remitting course fulfilling the 2010 McDonald criteria (Polman et al., 2011). To exclude the potential effects of relapse and medications on the brain function of the patients, all the participating patients had been relapse-free and were not treated with disease-modifying medications or steroids within 3 months before the MRI scans were obtained. We recruited 43 sex- and age-matched healthy controls (HCs; 30 females and 13 males) with no previous history of neurologic dysfunction and with normal findings from the neurologic examination and MRI.

### Clinical Assessment

The demographic and clinical characteristics, including disease duration, Expanded Disability Status Scale (EDSS) score (Kurtzke, 1983), Multiple Sclerosis Severity Score (MSSS; Roxburgh et al., 2005), and 2- and 3-s paced auditory serial addition tests (PASAT-2 and PASAT-3), were recorded by an experienced neurologist (HD, with more than 25 years of experience in neurology) at the time of the MRI.

### Image Acquisition

The MRI data were acquired on a 3.0-T MR system (Trio Tim; Siemens, Erlangen, Germany) with a 12-channel head coil. Resting-state fMRI data were collected using an echo planar imaging sequence, with 35 axial sections acquired: repetition time (TR)/echo time (TE) = 2,000/30 ms; flip angle = 90°; slice thickness = 3 mm; gap = 1 mm; in-plane resolution, 3.5 mm × 3.5 mm; and matrix size = 64 × 64. During resting-state fMRI, the subjects were instructed to keep their eyes closed, to remain motionless, and to not think of anything. High-resolution T_1_-weighted images were acquired with a magnetization-prepared rapid acquisition gradient-echo sequence [TR/TE = 1,600/2.13 ms, inversion time (TI) = 1,000 ms, flip angle = 9°, field of view (FOV) = 256 mm × 224 mm, matrix size = 256 × 224, slice thickness = 1.0 mm, voxel dimensions = 1.0 mm × 1.0 mm × 1.0 mm]. Axial T_2_-weighted turbo spin-echo (TR/TE = 5,000/87 ms, number of signals acquired = 1, echo train length = 15, FOV = 256 mm × 256 mm, matrix size = 256 × 256) was positioned parallel to a line that joined the most inferoanterior and inferoposterior parts of the corpus callosum, with number of slices of 30, 4-mm section thickness, and 0.4-mm intersection gap, and was used to measure the hyperintense brain lesions of MS patients and exclude HCs with brain lesions.

### Brain Lesion Analysis

Marking and measurement of hyperintense brain lesions [T_2_ lesion volume (T_2_-LV)] on T_2_-weighted images were performed by an experienced neuroradiologist (ZQ) by using MRIcro software^1^.

### Preprocessing of Functional MRI Data

Similar to a previous study (Ding et al., 2018; Li et al., 2019), the fMRI images were preprocessed using an in-house MATLAB script developed based on statistical parametric mapping (SPM12) software (Penny and Friston, 2003) and a toolkit for resting-state fMRI named Resting-State fMRI Data Analysis Toolkit (Song et al., 2011). The images were first corrected for slice timing and intervolume head motion. Then, the corrected images were coregistered to the native T_1_ image, which was then segmented into three probabilistic masks: GM, WM, and cerebrospinal fluid. Using a nonlinear normalization approach, the fMRI images, along with the anatomical masks, were subsequently transformed to a standard coordinate system called Montreal Neurological Institute (MNI) space. This procedure was reciprocal and provided forward (T_1_ space towards MNI) and backward (MNI towards T_1_ space) transformation matrices. The BOLD time series of each voxel of the normalized fMRI images was corrected for signal drift by removing the linear trends. Then, the signals were filtered with a bandpass filter (0.01–0.1 Hz) to remove physiological sources of noise by transforming the signals to the frequency domain using fast Fourier transform (FFT), masking out frequencies outside the filter range (0.01–0.1), and performing inverse FFT to transform signals back to the time domain. Finally, time series in the preprocessed BOLD images were normalized into unit variance in a voxelwise manner (Ding et al., 2018).

### Reconstruction of the Correlation Matrix

For each fMRI image, the GM and the WM were parcellated into multiple anatomical structures. Specifically, the GM was parcellated into 82 functional units based on Brodmann’s definitions (Maldjian et al., 2003) in MNI space, while the deep WM voxels were grouped into 48 tracts of interest using the JHU ICBM WM atlas (Mori et al., 2008), as shown in Supplementary Table e-1. In addition, all tracts were further constrained within the WM probabilistic mask of each subject and thresholded at a high value of 0.85. Average time series within each anatomical structure was obtained and then used to reconstruct the correlation matrix, in which each element denotes the Pearson’s correlation coefficient between one GM and one WM time series. All correlation coefficients were transformed to z-scores using Fisher’s Z transformation before statistical calculations so that the sampling distribution of the resulting variables is approximately normal.

### Quantitative Analysis of Brain Lesions

The lesion masks were first labeled by an experienced neuroradiologist (ZQ) in the T_2_ image space. Then, the T_2_ images combining lesion masks were registered to the T_1_ images. Finally, the lesion masks (lesion = 1, non-lesion = 0) were spatially wrapped to the MNI space using the forward (T_1_ space towards MNI) transformation matrices generated during the preprocessing. An accumulated lesion mask was obtained by performing a cumulative statistical analysis of the lesion mask of all patients. The normalized accumulated lesion mask is shown in Figure 4; the value of each pixel indicates the incidence rate among patients. We define a novel factor to quantitatively evaluate the degree of morbidity of each bundle among the patients.

**Figure 1:**
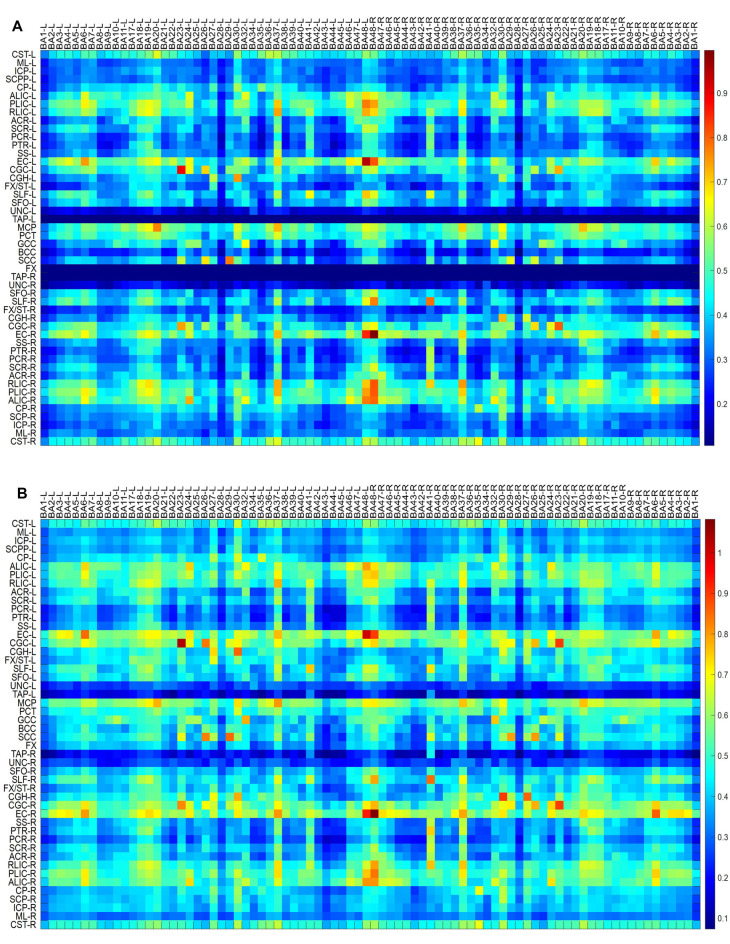

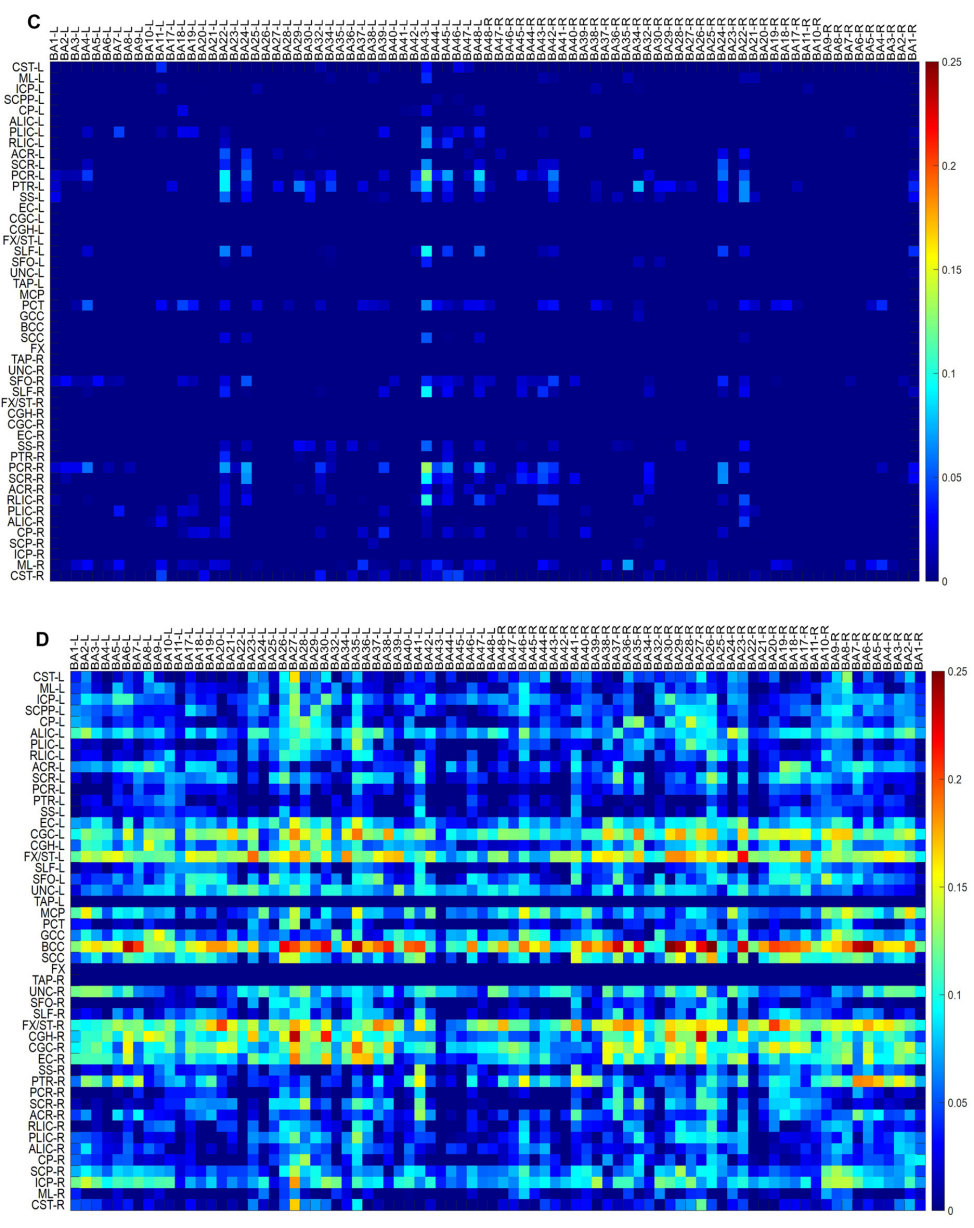
Maps of temporal correlations between blood oxygen level-dependent (BOLD) signals in white matter (WM) bundles (ordinate) and gray matter (GM) regions (abscissa). Top to bottom maps **(A–D)** show the data for multiple sclerosis (MS) patients, healthy controls, MS patients—healthy controls, and healthy controls—MS patients, respectively.

**Figure 2:**
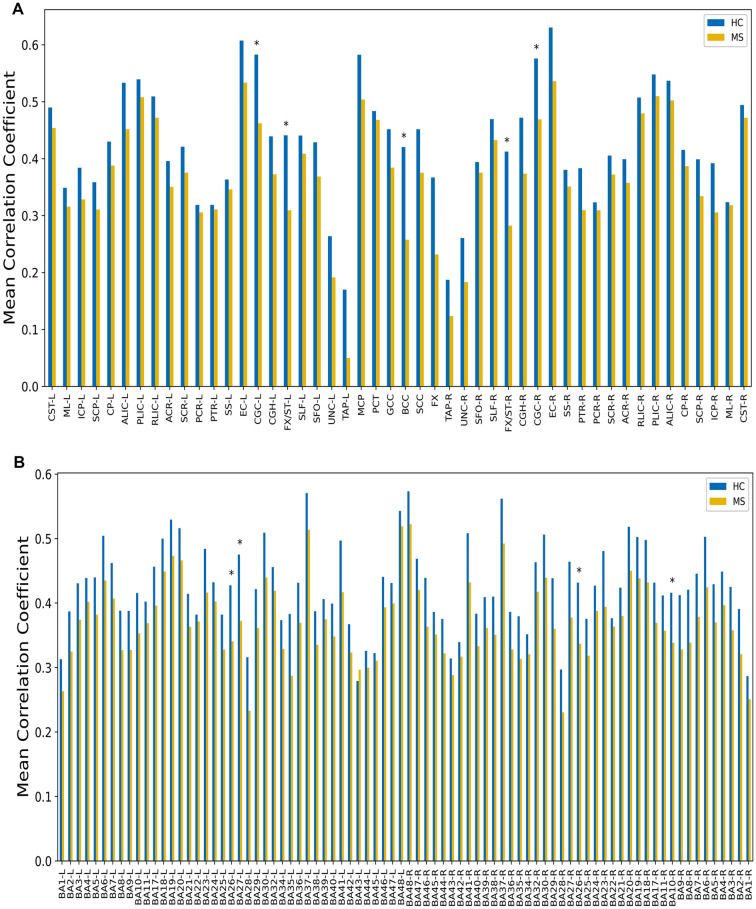
GM-averaged correlation coefficients (CCs) of WM **(A)** and WM-averaged CCs of GM **(B)**. Five of 48 WM bundles displayed a significantly decreased mean correlation coefficient [using two-sample *t*-test with a false positive-corrected *P < (1/48) = 0.021], while four of the 82 GM regions exhibited decreased mean CCs [using two-sample *t*-test with a false positive-corrected *P < (1/82) = 0.012].

**Figure 3:**
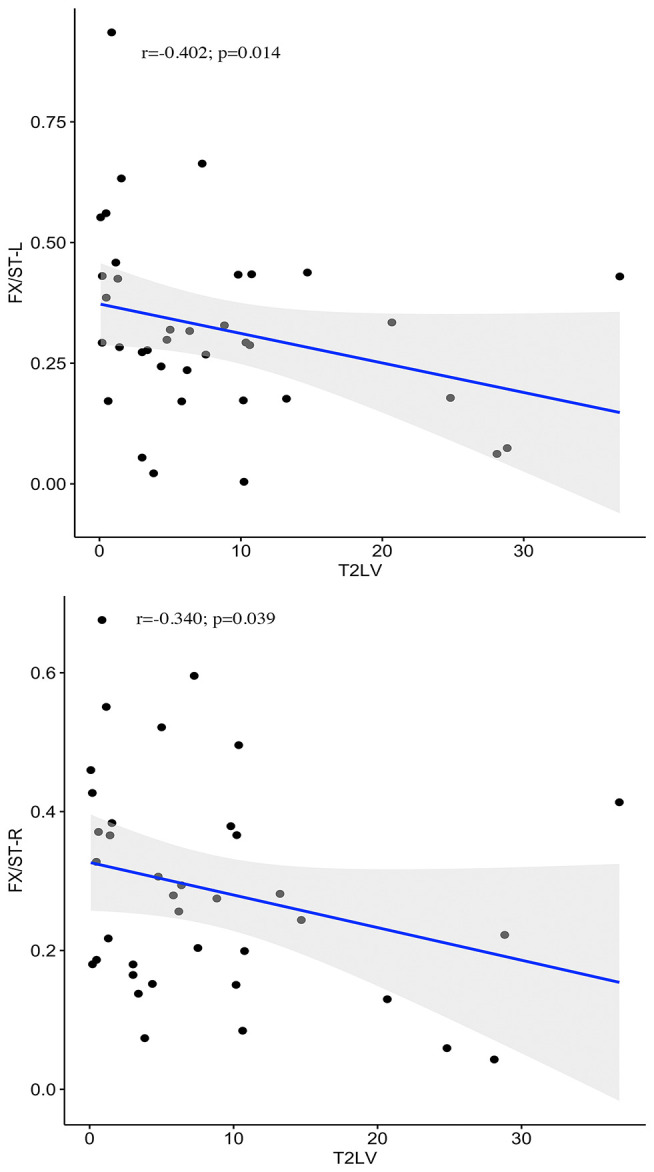
Correlations between WM functional alterations and T_2_ lesion volume. Negative correlations between the T_2_-LV and WM functional alterations in bilateral fornix (cres)/stria terminalis are presented in the graphs.

**Figure 4:**
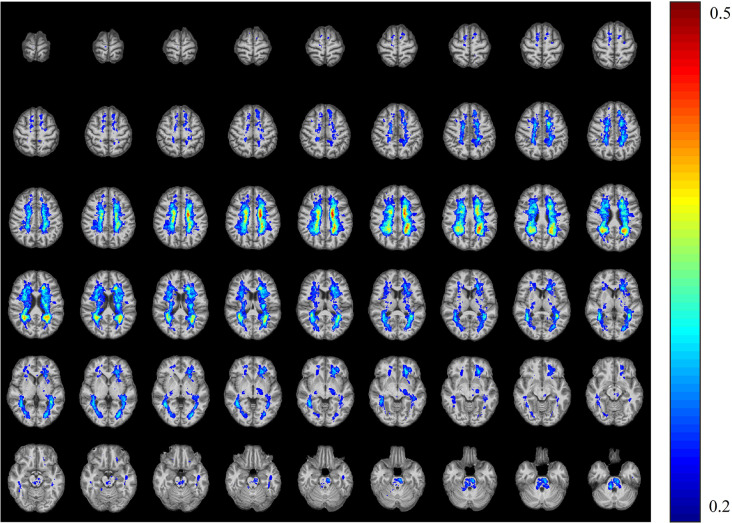
The T_2_WI lesion probability map in patients with multiple sclerosis. The color overlay was created on the ICBM152 T_1_ template in the Montreal Neurological Institute (MNI) standard brain. The mean lesion probability distribution thresholded at 50% is shown in dark red, and the probability distribution thresholded at 20% is shown in blue.

$$\text{lesion}\_{\text{ration}}_{i}=\frac{\sum_{x\in \text{bundle }i}\text{acc\_lesion\_value}\left(x\right)}{N_{i}}$$

where *x* represents the pixels that belong to each bundle, acc_lesion_value (*x*) is the accumulated lesion value of these pixels, and *N*_i_ is the total number of pixels in each bundle. A higher lesion_ratio value represents a higher degree of morbidity. We calculated the correlation between the lesion_ratio and the mean GM-averaged correlation coefficients (CCs) of the WM bundles.

### Statistical Analysis

The GM-averaged CCs of the WM bundles and the WM-averaged CCs of the GM regions were compared between the MS patients and HCs using a two-sample *t*-test, with a false-positive-corrected *P* < 1/48 for WM and *P* < 1/82 for GM being accepted as significant. Meanwhile, partial correlation analyses were performed to explore the potential relationship between these coefficients and the clinical characteristics of patients with MS [false discovery rate (FDR)-corrected].

### Data Availability

The data used to support the findings of this study are available from the corresponding author upon request.

## Results

### Demographic and Clinical Characteristics

The demographic characteristics of the participants are provided in Table 1. No significant differences in sex or age were observed between the two groups. All the MS patients had brain lesions, with an average T_2_-LV of 8.5 ml and an average disease duration of 42.7 months.

**Table 1:** Demographic and clinical characteristics.

Characteristics	MS (*n* = 37)	HC (*n* = 43)	*P*-values
Mean age (±SD; years)	34.6 ± 10.3	34.1 ± 11.0	0.893^a^
Sex (M/F)	11/27	13/30	0.722^b^
Median EDSS (range)	3.0 (0–6.5)	-	-
Median MSSS (range)	6.9 (0.8–9.5)	-	-
Median PASAT-2 (±SD)	35.3 (9.5)	-	-
Median PASAT-3 (±SD)	43.5 (9.4)	-	-
Median disease duration (±SD; months)	42.7 ± 34.3	-	-
Median T_2_-LV (±SD; ml)	8.5 (8.9)	-	-

*MS, multiple sclerosis; HC, healthy control; EDSS, Expanded Disability Status Scale; MSSS, Multiple Sclerosis Severity Score; PASAT, paced auditory serial addition test; T_2_-LV, T_2_ lesion volume. All subjects (patients with MS and HCs) were matched for age and sex. ^a^Two-sample *t*-test. ^b^Chi-square test*.

### Resting-State Correlations Between WM and GM in MS Patients

Maps of group-average temporal correlations between 48 WM bundles and 82 GM regions are shown in Figures 1A,B. Overall, the MS patients exhibited lower correlations than the HCs. The greatest differences between the MS patients and the HCs were identified in the temporal correlation of the body of the corpus callosum (BCC) and BA35-L, BA7-R, BA26-R, and BA30-R, which are intuitively observed because the patients with MS exhibited a lower temporal correlation than the HCs (Figures 1C,D). Figure 2 shows the GM-averaged CCs of the WM and WM-averaged CCs of the GM. Overall, MS decreased the temporal correlation between the WM and the GM regions. Our statistical comparisons showed significantly decreased mean CCs for five of the 48 WM bundles [two-sample *t*-test with a false-positive-corrected *P* < (1/48) = 0.021], which included the bilateral fornix (cres)/stria terminalis (FX/ST-L and FX/ST-R), bilateral cingulum (cingulate gyrus; CGC-L and CGC-R), and BCC. Four of the 82 GM regions displayed decreased mean CCs [two-sample *t*-test with a false-positive-corrected *P* < (1/82) = 0.012]; these regions were BA26-L, BA27-L, BA26-R, and BA10-R.

### Correlations Between Alterations in WM Functional Connectivity and Clinical Variables

No significant correlations between WM functional alterations and PASAT, EDSS, or MSSS scores were observed after FDR correction.

### Correlations Between Alterations in WM Functional Connectivity and the Lesion Load

Among the 48 WM bundles, two bundles, the bilateral FX/ST, exhibited significant negative correlations between the T_2_-LV and WM functional alterations (Figure 3). To further investigate the relationship between the frequency of lesion involvement and WM functional alterations, the correlations between the frequency of involvement of each WM bundle and alterations in the functional connectivity of that region were determined. As shown in Figure 6, the mean GM-averaged CCs of the WM bundles were inversely correlated with the lesion ratio (*r* = −0.36, *P* = 0.012).

**Figure 5:**
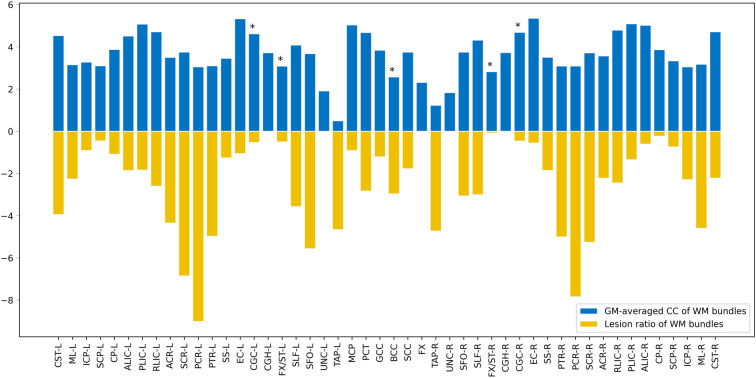
Map showing the comparison of WM bundle function and lesion ratio. The X axis depicts the 48 WM bundles, the positive longitudinal coordinate is the WM function of these bundles, and the negative longitudinal coordinate is the lesion ratio of the corresponding bundles. *P < (1/48) = 0.021.

**Figure 6:**
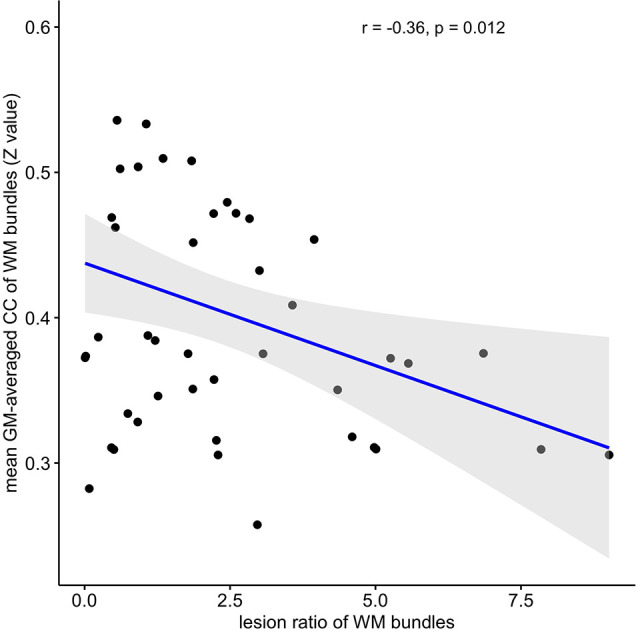
Correlation between WM functional alterations and the lesion ratio. The mean GM-averaged correlation coefficients of WM bundles were inversely correlated with the lesion ratio (*r* = −0.36, *P* = 0.012).

## Discussion

In the present study, we evaluated functional connectivity in both the WM bundles and the GM regions of MS patients using resting-state fMRI. The major findings of this study include the following:

(1) The patterns of temporal correlations in BOLD signals between segmented WM and GM regions were quite different between the MS patients and HCs. Overall, the correlation maps for the MS patients exhibited a much lower temporal CC than those for the HCs.
(2) The GM-averaged CCs in the WM bundles were generally decreased in patients with MS compared with HCs; most notably, the bilateral FX/ST, bilateral CGC, and BCC showed greater decreases than other WM bundles. Additionally, the WM-averaged CCs in four GM regions were significantly decreased in MS patients compared to HCs.
(3) WM functional alterations were associated with the WM lesion load but not with neurological or physical disabilities.

While fMRI has been established as a powerful neuroimaging tool for probing functional activity in the brain, to date, the vast majority of fMRI studies have focused on cortical GM, with BOLD signals in WM treated as noise or artifacts that are typically ignored. Therefore, regardless of the explosion of BOLD fMRI research, studies examining WM activation over the past two decades remain relatively scarce, with the exception of a limited number of task-based studies of WM function. Recently, WM functional activity has been observed during resting state in healthy human subjects (Ding et al., 2013, 2018) and patients with Parkinson’s disease (Ji et al., 2019). However, regarding MS, which is characterized by WM lesions as its cardinal pathological process, no studies have analyzed alterations in WM functional activity or connectivity. Therefore, an examination of WM function in patients with MS may improve the understanding, assessment, and diagnosis of disorders, in addition to extending brain activity research, and may yield new insights into the pathological changes in patients with these diseases.

In the present study, we examined functional connectivity within the WM by performing a correlation analysis of resting-state fMRI data. The matrix of the temporal correlations between WM bundles and GM regions showed that, compared to HCs, patients with MS exhibited a significantly decreased functional connectivity in widespread WM regions. Previous diffusion tensor imaging (DTI) studies (Bodini et al., 2009; Giorgio et al., 2010; Sbardella et al., 2013) have revealed that MS patients had significant alterations in diffusion profiles, such as widespread decreased fractional anisotropy and increased mean diffusivity in the WM, which are characteristic of the structural changes in MS. Interestingly, the pattern between functional connectivity obtained from fMRI and structural alterations characterized using DTI appears to be quite similar in WM. The reduced functional connectivity in WM tends to suggest compromised information transmission in these structures, which may be a direct cause of WM fiber bundle anomalies induced by the pathology of demyelination and axonal injury in MS patients. This study extended previous findings of widespread WM abnormalities in patients with MS from the perspective of WM dysfunction. At present, due to a lack of correlation analyses between the structure and the function of WM, further studies are warranted to reveal the mechanisms underlying these abnormal changes.

Regarding the GM-averaged CCs in the WM, greater decreases in WM functional connectivity were robustly identified within tracts of the limbic pathways (fornix and CGC) and callosal interhemispheric fibers (corpus callosum). Notably, these fibers, as parts of the limbic system, play a crucial role in cognitive deficits. The limbic system has often been shown to be affected in the MS process through magnetization transfer ratios and DTI studies pursuing an understanding of the neural correlates of abnormal cognitive symptomatology (Ranjeva et al., 2005; Dineen et al., 2009; Roosendaal et al., 2009; Fink et al., 2010; Kern et al., 2012; Syc et al., 2013; Huang et al., 2018; Keser et al., 2018). Miri et al. (2019) evaluated a group of task-related fMRI data with graph theory analysis and found that some parts of the limbic system in patients with MS were affected by cognitive impairment, which indicated the potential of graph theory analysis of task-related fMRI data to reflect early cognitive impairments in patients with MS. As shown in the present study, these bundles were functionally injured and, when combined with previous studies of structural damage, implied that these WM bundles suffered multidimensional impairment in patients with MS, i.e., simultaneous structural and functional damage, which may be a potential indicator for evaluating the cognitive impairment of patients with MS using resting-state fMRI.

Regarding the WM-averaged CCs in the GM, four of 82 GM regions displayed decreased mean CCs in patients with MS compared to the HCs. These findings attested to decreased functional connectivity of GM in the disease process, consistent with previous fMRI studies that observed cortical functional changes in patients with MS (Filippi et al., 2004; Rocca et al., 2005). In addition, our results are in line with the results of a recent study (Miri et al., 2019) that used task-state fMRI data to detect differences in functional connectivity networks between patients with MS and normal controls. The CCs between the WM and the GM might provide complementary information and thus potentially reveal the entirety of the functional changes in patients with MS.

To further understand the clinical significance of WM function in MS, we examined the associations of WM functional alterations with clinical variables and the WM lesion ratio. These clinical variables, including cognitive-related score (PASAT) and physical disability-related scores (EDSS and MSSS), are widely used to evaluate clinical disability and monitor disease progression in patients with MS. Previous studies have found that GM involvement, GM atrophy, and GM functional alterations were related to PASAT and EDSS scores (Filippi et al., 2013; Schoonheim et al., 2014; Eshaghi et al., 2018). In particular, supratentorial brain atrophy has displayed the strongest correlation with clinical disability (Rudick et al., 2009). It should be noted that, in the current study, no significant associations between alterations in WM function and the PASAT, or EDSS, and MSSS scores were observed. One possible explanation may be the small sample size of the study (only 37 MS patients). Longitudinal studies with larger sample sizes will be required to obtain a better understanding of the correlation between WM function and cognitive or physical disability levels. In addition, we observed negative correlations between T_2_-LV and WM functional alterations in two WM bundles. Taken together, these findings may have the following implications: first, WM function decreased with the increase in the WM lesion volume in MS patients; second, T_2_-LV was correlated with WM function, which may be due to the extent of WM bundle involvement determined by T_2_-LV, and the pathological changes due to inflammatory demyelination in the involved WM fibers in MS patients (Moore et al., 2000); abnormal changes in WM function were observed; third, among the two fiber tracts associated with T_2_-LV, the FX/ST-L (*r* = −0.402) was the tract that was most affected by the MS lesions, suggesting that the FX/ST-L may be sensitive bundles for observing WM functions in MS patients. The lesion ratio was negatively correlated with the mean GM-averaged CCs of the WM bundles with a moderate correlation strength (*r* = −0.36), and this correlation indicates that WM function decreases with the extent of WM bundle damage. Notably, the WM function of the fibers with the highest frequency of damage was not significantly reduced (see Figure 5); for example, the damage frequency of bilateral corona radiata was high, as these fiber bundles were involved in more than 25% of the patients (see Figure 4), but the function of these fiber bundles was not significantly reduced. We speculate that although the frequency of lesions in these bundles was high, the extent of the damage may not be serious, and thus the WM function was not significantly reduced. Therefore, in future studies, we should apply WM structural parameters (such as DTI) that assess the degree of WM fiber bundle damage to obtain a better understanding of the relationship between WM fiber bundle damage and its function and identify MRI biomarkers for monitoring damage to WM structure and function in patients with MS.

## Limitations

This study has some limitations. First, this retrospective cross-sectional study analyzed a relatively small sample, and a further longitudinal study with a larger sample size is warranted to validate the current findings. Second, MS is characterized by several pathological changes, such as focal WM lesions and diffuse WM occult injury, so-called normal appearing WM, GM lesions, GM atrophy, etc. However, this study only focuses on the relationship between focal WM lesions and WM function, and future studies should analyze the correlation between other pathological changes and WM function, which will provide a deeper understanding of the WM function in patients with MS. Third, we focused only on the WM functional changes; structural analyses, such as the use of a DTI-based method, were not performed in the present study. Furthermore, whether and how the WM functional abnormalities are related to structural abnormalities need to be examined, for instance, by integrating DTI and fMRI to assess the putative connection between structure and function. Additionally, the underlying mechanisms of MS should be better elucidated.

## Conclusions

In the current study, we demonstrated that WM bundles in MS patients exhibit decreased functional alterations and revealed the relationship between WM function and lesion characteristics. These findings improve our understanding of the disease from a new perspective of WM function.

## Data Availability Statement

The data analyzed in this study is subject to the following licenses/restrictions: the data used to support the findings of this study are available from the corresponding author upon request. Requests to access these datasets should be directed to sainthj@126.com.

## Ethics Statement

The studies involving human participants were reviewed and approved by the institutional review board of Xuanwu Hospital, Capital Medical University. The patients/participants provided their written informed consent to participate in this study.

## Author Contributions

ZD and JL designed the experiments. JH, ZQ, and KL performed the experiments. HD and ZL contributed to patient recruitment. ML, QL, ZY, and BX analyzed the sequencing data and developed the analytical tools. JH and ML drafted the manuscript. JH, ML, ZD, and JL initiated and supervised the entire project. All authors contributed to the article and approved the submitted version.

## Conflict of Interest

The authors declare that the research was conducted in the absence of any commercial or financial relationships that could be construed as a potential conflict of interest.

## Footnotes

^1^https://www.mccauslandcenter.sc.edu/crnl/mricro/

## Abbreviations

CC: correlation coefficient
EDSS: Expanded Disease Severity Scale
fMRI: functional MRI
GM: gray matter
MS: multiple sclerosis
MSSS: Multiple Sclerosis Severity Score
PASAT: paced auditory serial addition test
T2-LV: T2 lesion volume
WM: white matter.
