# Supplementary material for: Altered Functional Connectivity in White and Gray Matter in Patients With Multiple Sclerosis
Source: Front Hum Neurosci. 2020 Dec 2;14:563048. doi: 10.3389/fnhum.2020.563048 (PMC7738428; doi:10.3389/fnhum.2020.563048)
Supplement: Supplementary file 1 [file Table_1.DOCX]

**Supplementary tables:**

**Table e-1.** List of abbreviations for 48 WM bundles

| Abbreviation | Full name |
| --- | --- |
| CST-L | Corticospinal tract L |
| ML-L | Medial lemniscus L |
| ICP-L | Inferior cerebellar peduncle L |
| SCP-L | Superior cerebellar peduncle L |
| CP-L | Cerebral peduncle L |
| ALIC-L | Anterior limb of internal capsule L |
| PLIC-L | Posterior limb of internal capsule L |
| RLIC-L | Retrolenticular part of internal capsule L |
| ACR-L | Anterior corona radiata L |
| SCR-L | Superior corona radiata L |
| PCR-L | Posterior corona radiata L |
| PTR-L | Posterior thalamic radiation OR L |
| SS-L | Sagittal stratum L |
| EC-L | External capsule L |
| CGC-L | Cingulum (cingulate gyrus) L |
| CGH-L | Cingulum (hippocampus) L |
| FX/ST-L | Fornix (cres) / Stria terminalis L |
| SLF-L | Superior longitudinal fasciculus L |
| SFO-L | Superior fronto-occipital fasciculus L |
| UNC-L | Uncinate fasciculus L |
| TAP-L | Tapetum L |
| MCP | Middle cerebellar peduncle |
| PCT | Pontine crossing tract |
| GCC | Genu of corpus callosum |
| BCC | Body of corpus callosum |
| SCC | Splenium of corpus callosum |
| FX | Fornix (column and body of fornix) |
| TAP-R | Tapetum R |
| UNC-R | Uncinate fasciculus R |
| SFO-R | Superior fronto-occipital fasciculus R |
| SLF-R | Superior longitudinal fasciculus R |
| FX/ST-R | Fornix (cres) / Stria terminalis R |
| CGH-R | Cingulum (hippocampus) R |
| CGC-R | Cingulum (cingulate gyrus) R |
| EC-R | External capsule R |
| SS-R | Sagittal stratum R |
| PTR-R | Posterior thalamic radiation OR R |
| PCR-R | Posterior corona radiata R |
| SCR-R | Superior corona radiata R |
| ACR-R | Anterior corona radiata R |
| RLIC-R | Retrolenticular part of internal capsule R |
| PLIC-R | Posterior limb of internal capsule R |
| ALIC-R | Anterior limb of internal capsule R |
| CP-R | Cerebral peduncle R |
| SCP-R | Superior cerebellar peduncle R |
| ICP-R | Inferior cerebellar peduncle R |
| ML-R | Medial lemniscus R |
| CST-R | Corticospinal tract R |
